# Information-seeking in mice (*Mus musculus*) during visual discrimination: study using a distractor elimination paradigm

**DOI:** 10.1007/s10071-024-01920-3

**Published:** 2024-12-02

**Authors:** Yuya Hataji, Kazuhiro Goto

**Affiliations:** 1 Keio University, Tokyo, Japan; 2 Japan Society for the Promotion of Science, Tokyo, Japan; 3 Sagami Women’s University, Kanagawa, Japan

**Keywords:** Metacognition, Information-seeking, Cognitive control, Visual discrimination, Rodents, Touchscreen

## Abstract

Some animals seek information to solve problems when they do not know the answer. Information-seeking behavior has become a key focus in studies of animal metacognition, providing insights into how animals monitor their own knowledge states. This behavior is thought to be a form of metacognitive control. Nevertheless, research on such metacognitive control has been biased toward specific taxa, such as primates, and has not been conducted in rodents, which are the most common experimental animals. This study examined whether mice exhibit information-seeking behavior during two visual discrimination tasks and what factors influence this behavior. We trained mice to discriminate between stimuli differing in luminance or orientation, with more minor differences increasing task difficulty. An information-seeking option was introduced during these tasks, allowing mice to eliminate distractor stimuli and ensure a correct response. The results indicated that mice sought information more frequently during difficult discriminations than easier ones. However, subsequent generalization tests revealed that the mice relied on environmental cues to utilize the information-seeking option. These findings suggest that information-seeking behavior in mice may not solely reflect metacognitive processes, and further investigation is needed to explore alternative explanations.

**Supplementary Information:**

The online version contains supplementary material available at 10.1007/s10071-024-01920-3.

## Introduction

Metacognition plays a crucial role in decision-making and problem-solving in everyday life. For example, if you find yourself locked out of an account after forgetting your password, your first instinct might be to recall the password by trying various possibilities. However, recognizing the challenge of remembering passwords, a more adaptive strategy to avoid forgetting passwords is to seek out a password management application. This behavior reflects an awareness of your own knowledge limitations and the ability to seek information to solve problems, thereby demonstrating metacognitive control.

Information-seeking behavior, as a form of metacognitive control, is not exclusive to humans; it has also been observed in other species. Call and Carpenter (2001) devised the tube task as a pivotal experimental paradigm for investigating animal information-seeking behavior. Their study presented chimpanzees and orangutans with tasks in which they hid food into one of several tubes. The apes could either see where the food was baited or had to locate it themselves. They looked into the tubes more frequently when they did not observe where the food was hidden compared to when they did. Similar behaviors have been observed across different species. For example, orangutans (Marsh and McDonald 2012), gorillas (Gazes et al. 2023), rhesus monkeys (Hampton et al. 2004), and Japanese macaques (Subias et al. 2024) exhibit information-seeking behavior under similar conditions. These findings suggest that the ability to seek information in uncertain situations may be a shared trait among primate species.

However, not all animals exhibit the same behavior. For instance, lion-tailed macaques (Marsh et al. 2014), capuchin monkeys (Basile et al. 2009; Paukner et al. 2006), and lemurs (Taylor et al. 2020) seek information regardless of whether they have observed where the food is hidden. Interestingly, this redundant information-seeking behavior is also found in non-primate species, such as ravens (Lambert and Osvath 2020).

While the tube task is an elegant approach, it has significant limitations for advancing the study of comparative metacognition. A primary limitation is that it assumes the subject animal can understand and remember the location of the food after observing the experimenter hide it. As a result, the application of the task is restricted to animals that are sensitive to human actions and capable of comprehending the consequences of those actions. Additionally, alternative explanations, which are more parsimonious than a metacognitive interpretation, can account for the observed behavior of looking into the tube (Carruthers 2008; Crystal and Foote 2011; Roberts et al. 2012). For instance, looking into the tube might be explained by response competition, where reaching into the tube is a more robust response after seeing the food, while merely looking into the tube is a weaker, secondary response. Although Call (2010) addressed this response competition account by manipulating the quality of rewards, these limitations generally highlight the need for novel tasks to study information-seeking behavior in species that are challenging to assess using the tube task.

Researchers have developed several computer-based tasks for primates to address these limitations (e.g., Basile et al. 2015; Brady and Hampton 2021; Kornell et al. 2007; Malassis et al. 2015; Tu et al. 2015). These tasks have replicated and reinforced the findings from the tube task, in which rhesus monkeys exhibit selective information-seeking behavior, while capuchin monkeys display redundant information-seeking (Beran and Smith 2011).

Interestingly, pigeons are the only species outside primates that can demonstrate information-seeking behavior using a computer-based task. Iwasaki et al. (2013) investigated hint-seeking behavior in pigeons while learning response sequences to four icons. Two of the four pigeons in this study showed higher rates of hint-seeking during the early stages of learning a new sequence compared to later stages. However, this behavior did not transfer to a visual search task, suggesting that the pigeons’ control over their behavior based on their knowledge state was inconclusive.

In contrast, Castro and Wasserman (2013) reported that pigeons could transfer information-seeking behavior trained in one task to another. They trained pigeons on a conditional same-different discrimination task involving visual arrays of varying difficulty, incorporating an “information” button that allowed them to ease the task by increasing the number of items in one array. A “Go” button enabled the pigeons to proceed without seeking additional information. The results showed that as task difficulty increased, pigeons used the “information” button more frequently, demonstrating adaptive behavior to reduce the difficulty before making a discrimination response. Moreover, some pigeons successfully transferred this behavior to new tasks involving luminance and size discriminations.

Although touchscreen chambers have become widely used in rodent research (Sullivan 2022), computer-based information-seeking tasks have not yet been developed. Developing such tasks is crucial, as rodents are essential laboratory animals for translational research. Some studies have reported metacognitive abilities in rats (Templer 2019). For example, Templer et al. (2017) reported that rats performed better on trials in which they could opt out of the test than when they were forced to complete a delayed matching-to-sample olfactory task. Additionally, rats declined memory tests when the delay was long or no sample was presented. Similarly, Yuki and Okanoya (2017) reported that rats declined memory tests; however, in their study, the decline response was adaptive only when the memory test was difficult, and the results were less robust than those reported by Templer et al. (2017).

Several studies have investigated metacognitive abilities in rats. However, researchers have made little progress in specifically studying information-seeking behavior. Even less is known about these functions in mice, particularly in the context of information-seeking during cognitive tasks. The present study investigated the discriminative stimuli that control information-seeking behavior during visual discrimination in mice to address this knowledge gap. We presented an information-seeking option during a two-alternative forced-choice visual discrimination task. The reward did not directly reinforce the information-seeking behavior. However, removing the distractor stimulus allowed the mice to select the target stimulus reliably in that trial.

We conducted five experiments to investigate information-seeking behavior in visual discrimination tasks. In experiment 1, we trained mice to respond to stimuli with different luminance levels on a black background to determine the range for subsequent luminance discrimination tasks. We hypothesized that mice would have difficulty responding to stimuli as their luminance approached the background color. Experiment 2 used pairs of stimuli with varying luminance levels to investigate whether mice seek information during luminance discrimination and what cues they use in this behavior. Based on the theoretical framework of metacognition, we hypothesized that mice would seek information more frequently in situations where uncertainty about the correct response was higher. We predicted that mice would use luminance differences between stimuli as a cue to guide their information-seeking behavior. Experiment 3 examined information-seeking behavior in a different type of visual discrimination task, in which mice identified the more horizontal or vertical of two line segments. We varied the task’s difficulty by manipulating the orientation difference between the line segments. We predicted that mice would use orientation differences between stimuli as a cue to guide their information-seeking behavior. In experiment 4, we explored whether increasing the vertical position of the stimulus pairs in the primary discrimination task in an attempt to introduce a response cost affected the frequency of information-seeking behavior. We hypothesized that increasing task demand by adding a physical response cost (standing higher to respond) would influence the frequency of information-seeking behavior. Specifically, we predicted that mice would be more likely to seek information when task demand was higher, as the overall benefit of seeking information would increase. Finally, in experiment 5, we further examined the effect of response cost by manipulating the number of choice alternatives in the primary discrimination task. We hypothesized that increasing the number of choice alternatives would increase task difficulty and, in turn, increase the rate of information-seeking behavior. We predicted that the mice would seek more information in tasks with more choice alternatives, as they would need to reduce uncertainty about their decisions.

## General methods

### Subjects

Six male C57BL/6N mice, obtained from Japan SLC, were transferred to the Sagami Women’s University animal facility at eight weeks of age. The mice were group-housed in cages with paper bedding (Shepherd Specialty Papers, Alpha-Dri) and maintained on a 12-hour light/dark cycle (lights on from 6:00 to 18:00). After a two-week acclimatization period, the mice were placed on a restricted diet. Their body weights were 23.7 g (range: 22.6–24.6 g) before dietary restrictions and 20.7 g (range: 19.3–21.6 g) after restrictions. Weight was monitored and maintained by supplementary feeding (typically 1.75 g per animal per day) in addition to the food rewards earned during daily testing sessions. Water was freely available in the cages, and all experiments were conducted during the light phase of the cycle. Cages were cleaned, and bedding was changed weekly to maintain a hygienic environment.

### Apparatus

**Fig. 1:**
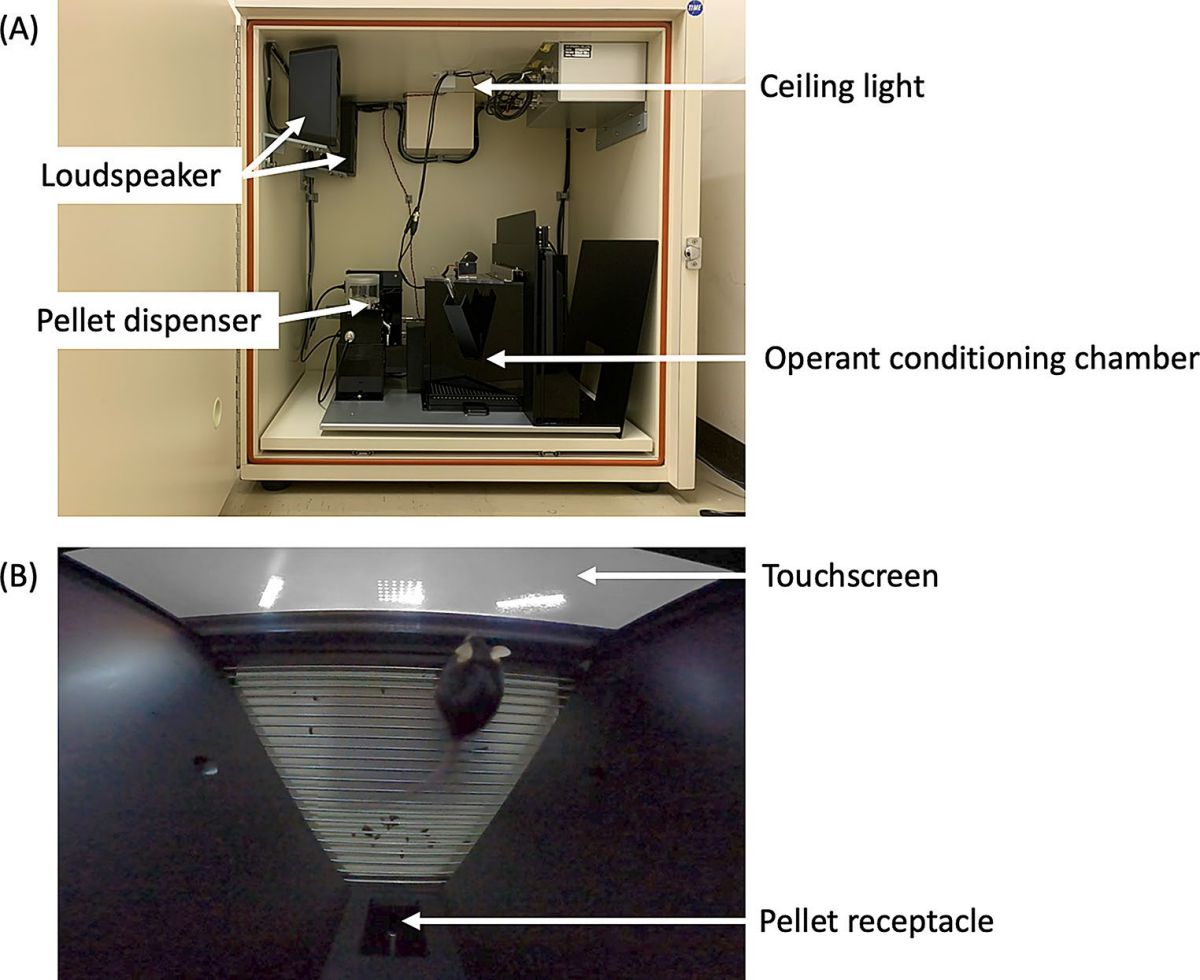
Operant conditioning chamber. The chamber is housed inside a sound-attenuating box, with all experimental procedures remotely controlled from a PC outside the chamber. (**a**) Side view of the apparatus. (**b**) Top view of the apparatus. This photo was taken from a video clip of the information-seeking trials during the orientation discrimination task in experiment 3

All experiments were conducted in an operant conditioning chamber (TOP-3002, O’hara & CO., Ltd.) housed within a sound-attenuating cubicle (TOP-4012N, O’hara & CO., Ltd.; Fig. 1). The chamber was trapezoidal (20 cm height × 18 cm length × 24 cm at the wide end, tapering to 6 cm at the narrow end) and featured three black acrylic walls. One wall housed a 15-inch monitor (K-155A, Eyoyo) with an infrared touch sensor (ARTS-015N-01B, Minato Advanced Technologies, Inc.). A black removable screen mask was placed over the monitor, leaving a 10 cm × 24 cm window through which the mice were trained to touch the screen. The bottom edge of the screen mask window was positioned 2.5 cm above the chamber floor.

A house light mounted at the top of the box remained illuminated throughout the session, and two loudspeakers (ST-SP93BK, Audiotechnica) located at the top rear of the box presented auditory stimuli. A pellet receptacle was attached to the chamber’s back wall, with a pellet dispenser (PD-010, O’hara & CO., Ltd.) located outside the chamber to deliver 10 mg food pellets (5TUL, TestDiet). The pellet receptacle was equipped with photocell detectors to detect mouse head entries and was illuminated by an LED that blinked at 250 ms intervals to signal the start of the trial. The light remained lit for 5 s when food was presented. The magazine light, dispenser, and head-entry detection were controlled via digital I/O (DAQ, National Instruments) connected to a nearby computer (Lenovo, ThinkPad L570). All system components were managed using custom software written in Python, utilizing PsychoPy version 3.2.4 (Peirce et al. 2019).

### Statistical analysis

All statistical analyses were conducted using R (version 4.4.1; R Core Team 2024). The tidyverse package (Wickham et al. 2019) was utilized for data processing and visualization. The lme4 package (Bates et al. 2015) was used to model the proportion of correct responses or information seeking responses using a generalized linear mixed model (GLMM) with a binomial distribution and logit link function via the Laplace approximation. Fixed effects and interactions were evaluated using a Type II Wald chi-square test. Fixed effects varied across the experiments (see Results and Discussion of each experiment). Subject ID was used as a random factor. Multiple comparisons were conducted using Tukey’s method of multcomp package (Hothorn et al., 2008).

## Experiment 1: determining the luminance threshold

This experiment aimed to determine the luminance threshold for use in a subsequent discrimination task. Mice were trained to touch a stimulus that varied in luminance relative to the background. Correct responses were reinforced with a food reward, whereas incorrect ones resulted in a time-out. The luminance threshold was when the proportion of correct responses fell to chance level.

## Methods

### Procedure

Before the threshold test, mice underwent habituation and magazine training in the operant conditioning chamber. Following this, they were trained to nose-touch a white circle (2.5 cm in diameter) centrally presented at the bottom of the monitor. Once the nose-touch response was acquired, stimuli were randomly presented in different horizontal positions on each trial. A self-start signal was introduced, and each trial began with the magazine light flashing at 250-ms intervals. The trial officially started when the mouse’s head entered the magazine. Previous studies have provided detailed training protocols (Goto and Hataji 2020; Goto and Watanabe 2021).

#### Threshold test

**Fig. 2:**
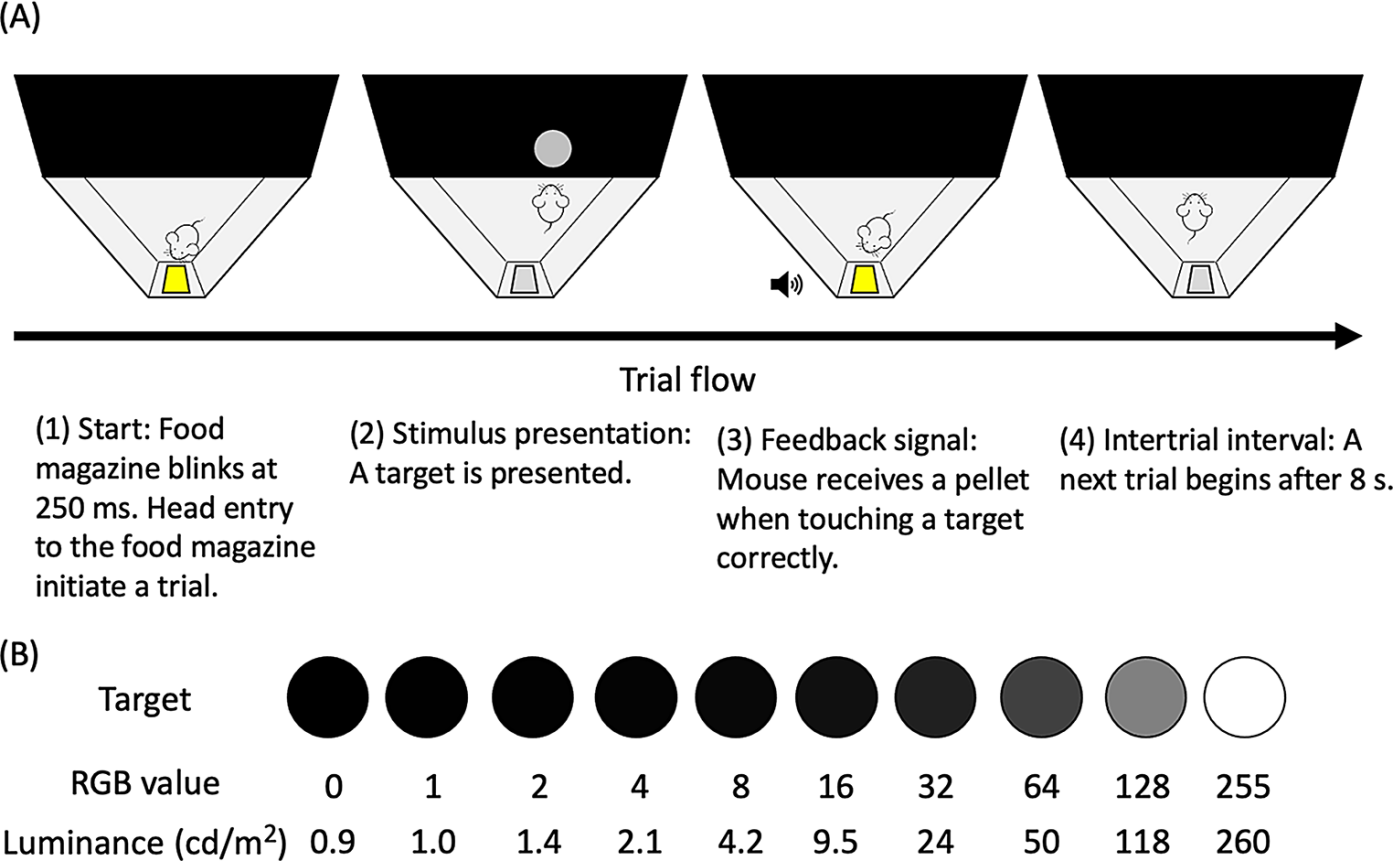
Procedures and target stimuli for the luminance detection task. (**A**) The trial began with the flashing of the magazine light, signaling the start. The target stimulus was presented on the monitor when the mouse placed its head into the magazine. Touching the target stimulus was reinforced with a 10 mg pellet. Other responses were not reinforced. The intertrial interval was 8 s. (**B**) The target stimuli consisted of ten different luminance levels, ranging from black to white, including shades of gray

Figure 2a illustrates the trial sequence and target stimuli used in the threshold test. Each trial commenced with the flashing of the magazine light, which turned off when the mouse placed its head in the magazine. A circle (2.5 cm in diameter) was then presented randomly at the bottom of the monitor. Touching the circle was reinforced with a food pellet, a 1-s chime, and a 5-s illumination of the magazine. Conversely, touches to any other monitor region were considered incorrect responses, resulting in the trial ending without reinforcement and a 5-s blackout. The intertrial interval was 8 s.

The luminance of the target stimulus was randomly selected from one of 10 levels within the RGB value range (255, 128, 64, 32, 16, 8, 4, 2, 1, and 0) against a background luminance of 0 and a maximum luminance of 255 (Fig. 2b). The corresponding measured luminance values were 260 cd/m², 118 cd/m², 50 cd/m², 24 cd/m², 9.5 cd/m², 4.2 cd/m², 2.1 cd/m², 1.4 cd/m², 1.0 cd/m², and 0.9 cd/m², respectively. Each session consisted of 60 trials, with six blocks of 10 luminance levels repeated randomly. Training continued five days per week until ten sessions were completed.

## Results and discussion

**Fig. 3:**
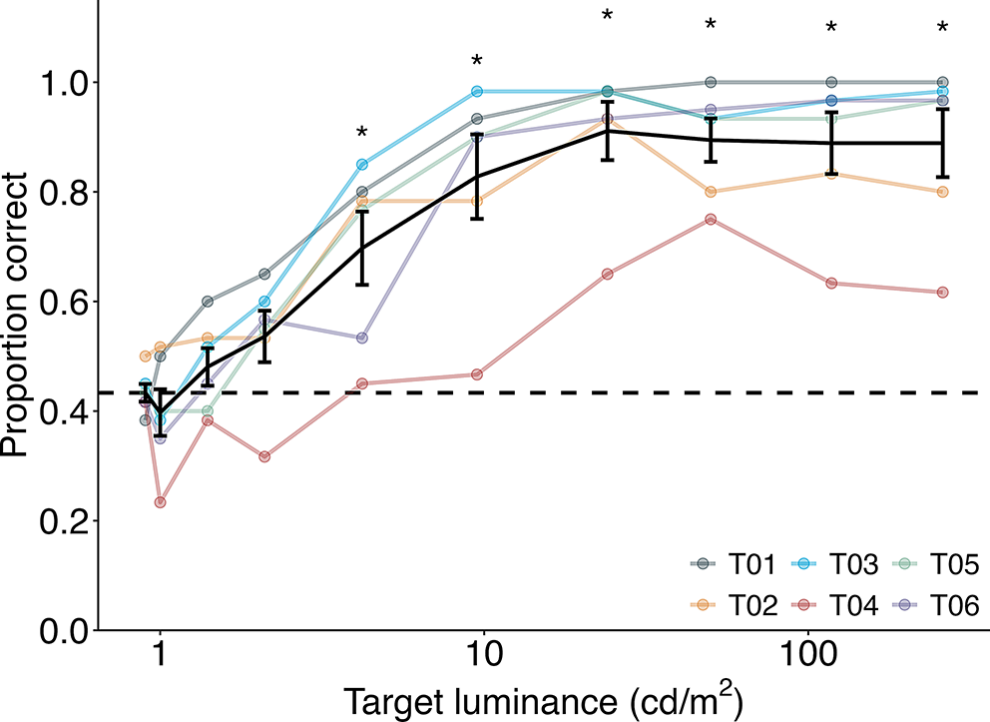
Luminance threshold. The black plot represents the mean proportion correct, whereas the colored plots represent individual performances. T01 to T06 are subject identifiers. Plots marked with an asterisk indicate performance significantly above the chance level (proportion correct when the target luminance was 0.9 cd/m², represented by the horizontal dashed line). Error bars indicate the standard error of the mean

All mice completed all stages of pre-training and progressed to the threshold test. Figure 3 illustrates the correct detection of the target stimulus as a function of its relative luminance to the background. A GLMM was fitted to the proportion of correct responses with target luminance as a fixed factor and subject as a random factor. The analysis confirmed a significant main effect of target luminance (χ^2^(9) = 601.2, *p* <.001). The proportion of correct responses was approximately 0.9 when the target luminance was 9.5 cd/m² or higher, but it gradually decreased to 0.4 when the target luminance matched the background luminance (0.9 cd/m²), indicating chance-level performance. Multiple comparisons between the luminance of 0.9 cd/m² (same as the background) and the other nine conditions revealed that the proportion of correct responses was significantly above chance when the target luminance was 4.2 cd/m² or higher (Table 1). Therefore, the lowest detectable luminance for the mice in this experiment was 4.2 cd/m².

**Table 1:** Results of multiple comparisons of proportion correct between target luminances

Luminance pair	z values	adjusted *p* values
1–0.9	-1.01	0.99
1.4–0.9	1.31	0.95
2.1–0.9	2.84	0.12
4.2–0.9	7.29	< 0.01
9.5–0.9	10.83	< 0.01
24 − 0.9	12.57	< 0.01
50 − 0.9	12.32	< 0.01
118 − 0.9	12.22	< 0.01
260 − 0.9	12.22	< 0.01

Two mice, T02 and T04, exhibited lower accuracy in touching the stimulus than the others. These mice often touched the target with their hindquarters while looking back at the food magazine on the back wall. Although this behavior was problematic, it was anticipated that it would be resolved in the following experiment, in which the training procedure required the mice to touch one of two relatively small stimulus regions and ignore touches to other areas. Consequently, training was continued without implementing specific behavioral modifications at this stage.

## Experiment 2: information-seeking behavior in luminance discrimination

Experiment 2 explored whether and how mice seek information during a luminance discrimination task. Building on the results from experiment 1, we developed a set of stimuli that varied in discrimination difficulty across the range of detectable luminances. We then assessed whether the mice’s information-seeking behavior was influenced by task difficulty by incorporating information-seeking options into the luminance discrimination task. Finally, we examined the mice’s information-seeking behavior in novel situations to understand the underlying mechanisms further.

## Method

### Stimuli

The stimuli consisted of two 2.5 cm squares with varying luminance levels. Based on the detection thresholds established in experiment 1, five luminance levels (200, 150, 113, 84, and 63) were selected, with a background luminance of 0 and a maximum luminance of 255. Ten pairs of stimuli were created, combining four different target stimuli (200, 150, 113, and 84) with four distractor stimuli (150, 113, 84, and 63).

**Fig. 4:**
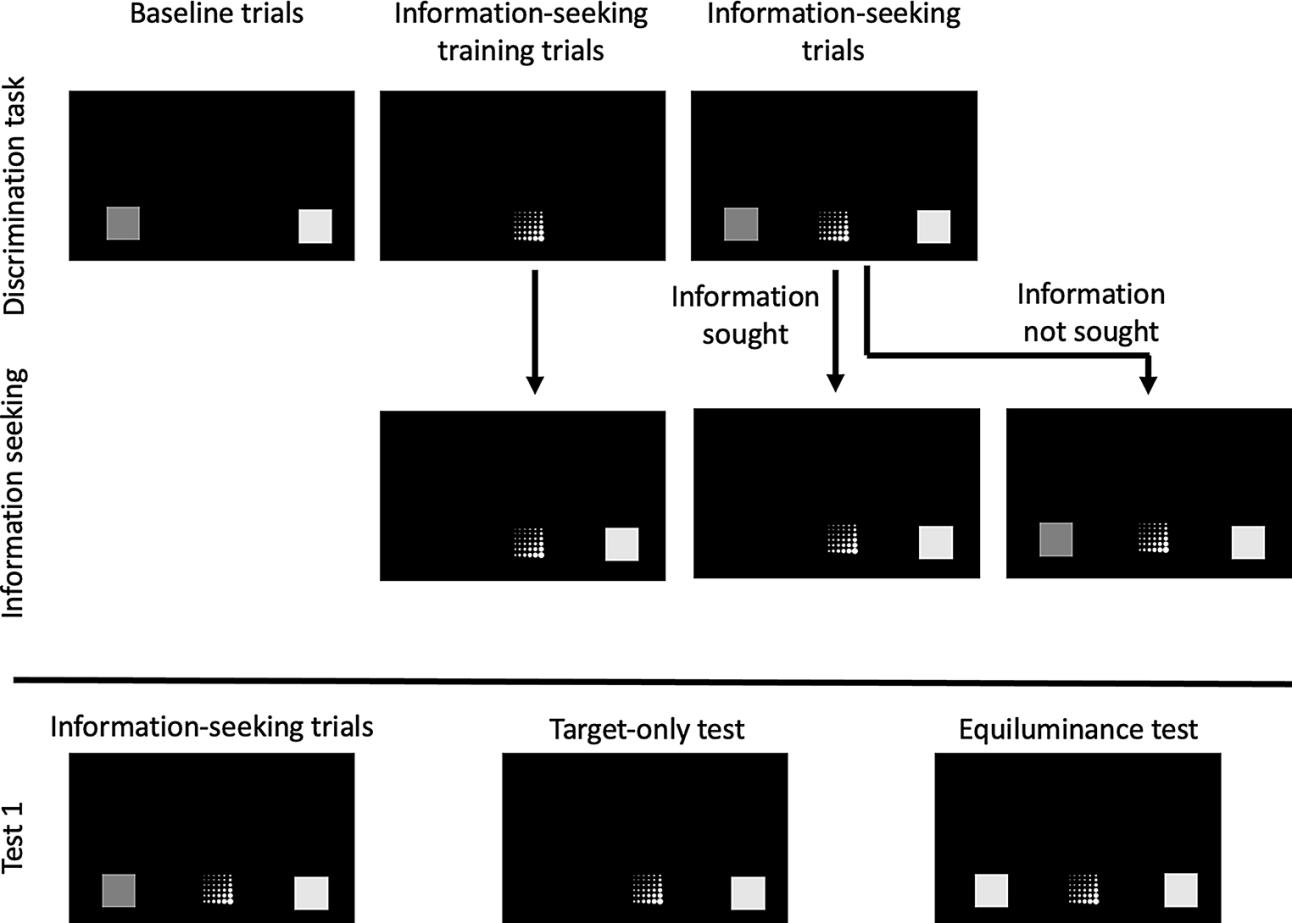
Types of training and test trials in Experiment 2. The upper panel illustrates the three types of luminance discrimination training trials. In baseline trials, two stimuli of different luminance were presented, with the brighter stimulus as the target. An information-seeking stimulus was presented during information-seeking training trials. If the subject responded to this stimulus, only the target stimulus remained on the screen. In information-seeking trials, two stimuli of different luminance were presented along with the information-seeking stimulus. If the subject chose the information-seeking stimulus, the distracting stimulus was removed, leaving only the target stimulus. Mice could also discriminate luminance directly without responding to the information-seeking stimulus

In some trial types, a 2.5 cm textured square composed of white dots was presented as an information-seeking option (Fig. 4, center stimulus in each panel). Mean luminance of the information-seeking option was 61.0 in RGB value.

### Procedure

During the luminance discrimination phase, each trial began with the flashing of the magazine light, which turned off when the mouse placed its head in the magazine. Following this, two 2.5 cm squares of different luminance were displayed horizontally on the monitor, separated by 12.5 cm. Mice were reinforced with a food pellet for choosing the brighter square while choosing the darker square resulted in a 5-second time-out. Incorrect choices triggered a correction trial, which was excluded from the analysis. The intertrial interval was 8 s, and each daily session comprised 60 randomly intermixed trials, equally divided among the ten stimulus pairs. This phase of luminance discrimination was conducted 5 days per week over 26 sessions.

After completing the luminance discrimination phase, an information-seeking option was introduced (Fig. 4). Each session included three types of trials: information-seeking training trials, information-seeking trials, and baseline trials. During the information-seeking training trials, the magazine light flashed and turned off when the mouse placed its head in it. An information-seeking option then appeared at the bottom center of the monitor. If the mouse selected this option, it remained on the monitor while the target stimulus appeared 6.25 cm to the left or right of the information-seeking option. No additional costs were incurred beyond those associated with responding to the option.

In the information-seeking trials, the magazine light turned off after the mouse’s head entered the magazine, and the information-seeking option appeared alongside the luminance discrimination task. Selecting the information-seeking option caused the distractor stimulus to disappear, leaving only the target stimulus on the screen. If the mouse did not select the information-seeking option, it proceeded with the luminance discrimination task as usual. Baseline trials involved the standard luminance discrimination task without the information-seeking option. Correct responses were reinforced with a pellet, while incorrect responses resulted in a 5-second time-out with the house light turned off. The stimulus pairs and target locations were randomized and counterbalanced trial-by-trial. Each session consisted of 16 information-seeking training trials, 20 information-seeking trials, and 20 baseline trials for 56 trials. This training phase lasted for 22 sessions.

### Test 1

In test 1, the mice were presented with baseline and information-seeking trials and two types of novel trials: Target-Only (TO) trials and Equiluminant (EL) trials. In TO trials, only the target stimulus was presented on either side of the information-seeking option, whereas in EL trials, two stimuli of the same luminance were presented, with one designated as the target. Each session consisted of 8 TO trials, 20 information-seeking trials, 8 EL trials, and 20 baseline trials, totaling 56 trials per session. This phase lasted for ten sessions.

### Test 2

Test 2 started the day after test 1. In test 2, we examined whether the absolute luminance of the target stimuli served as a cue for information-seeking. Novel stimulus pairs with approximately the same target-to-distractor luminance ratio were introduced using four pairs of RGB values: 9/39, 63/39, 63/49, and 255/200. The discrimination difficulty was consistent across all pairs. If the information-seeking behavior were based on metacognition, the rate of information-seeking should not vary across these test trials. Each session included 32 baseline trials and 32 test trials using the novel stimulus pairs, and this phase lasted for 9 sessions.

## Results and discussion

### Luminance discrimination

**Fig. 5:**
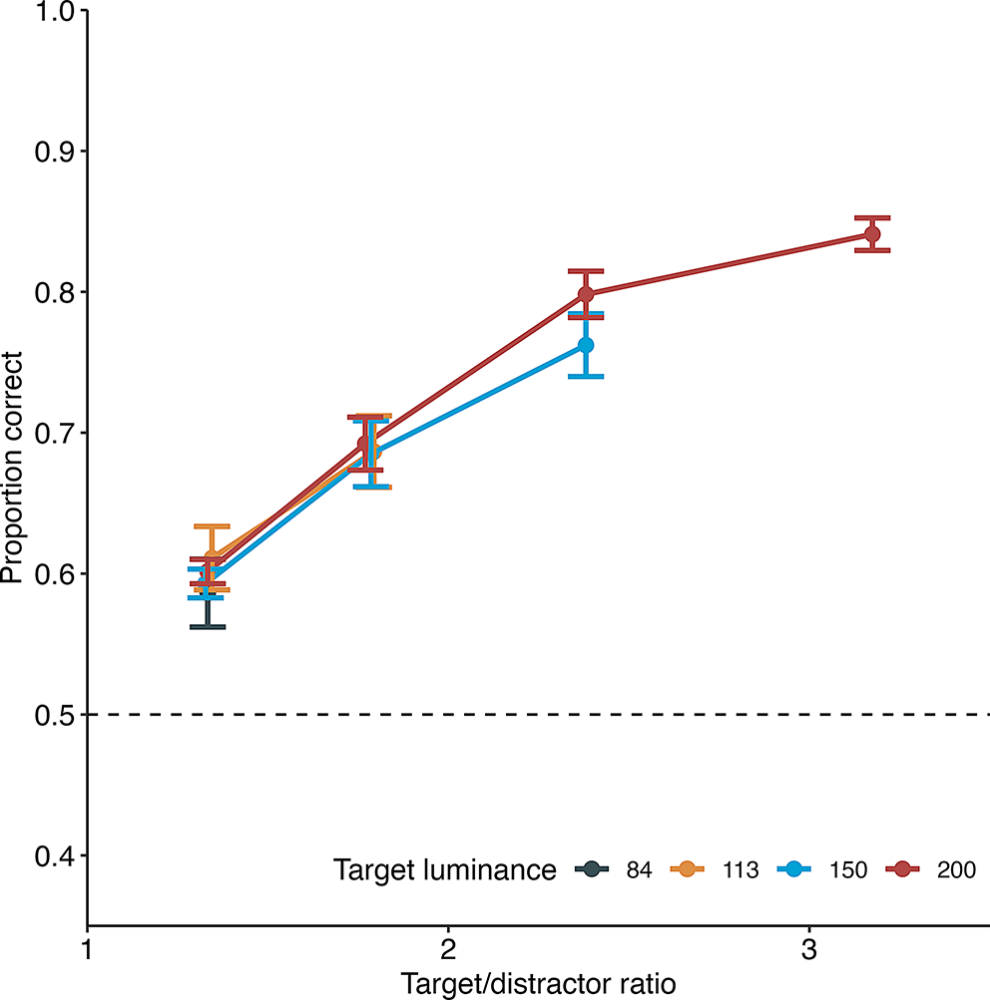
Luminance discrimination in experiment 2. The horizontal axis represents the ratio of target to distractor luminance. Error bars indicate the standard error of the mean. Individual data are shown in supplementary file Fig. 1S

Figure 5 presents the proportion of correct responses during the luminance discrimination task during 26 sessions. The data indicate that the proportion of correct responses increased as the luminance ratio between the target and distractor stimuli increased. A GLMM was applied with target luminance, luminance ratio, and their interaction as fixed factors and subjects as a random factor. The analysis revealed a significant main effect of luminance ratio (χ^2^(1) = 218.3, *p* <.001). However, neither the main effect of target luminance (χ^2^(1) = 1.2, *p* =.27) nor the interaction between luminance ratio and target luminance was significant (χ^2^(1) = 0.1, *p* =.7).

These findings suggest that varying luminance differences can effectively manipulate task difficulty. Consequently, in the subsequent test, luminance discrimination was utilized as the primary task to assess information-seeking behavior in mice.

Information-Seeking Training.

**Fig. 6:**
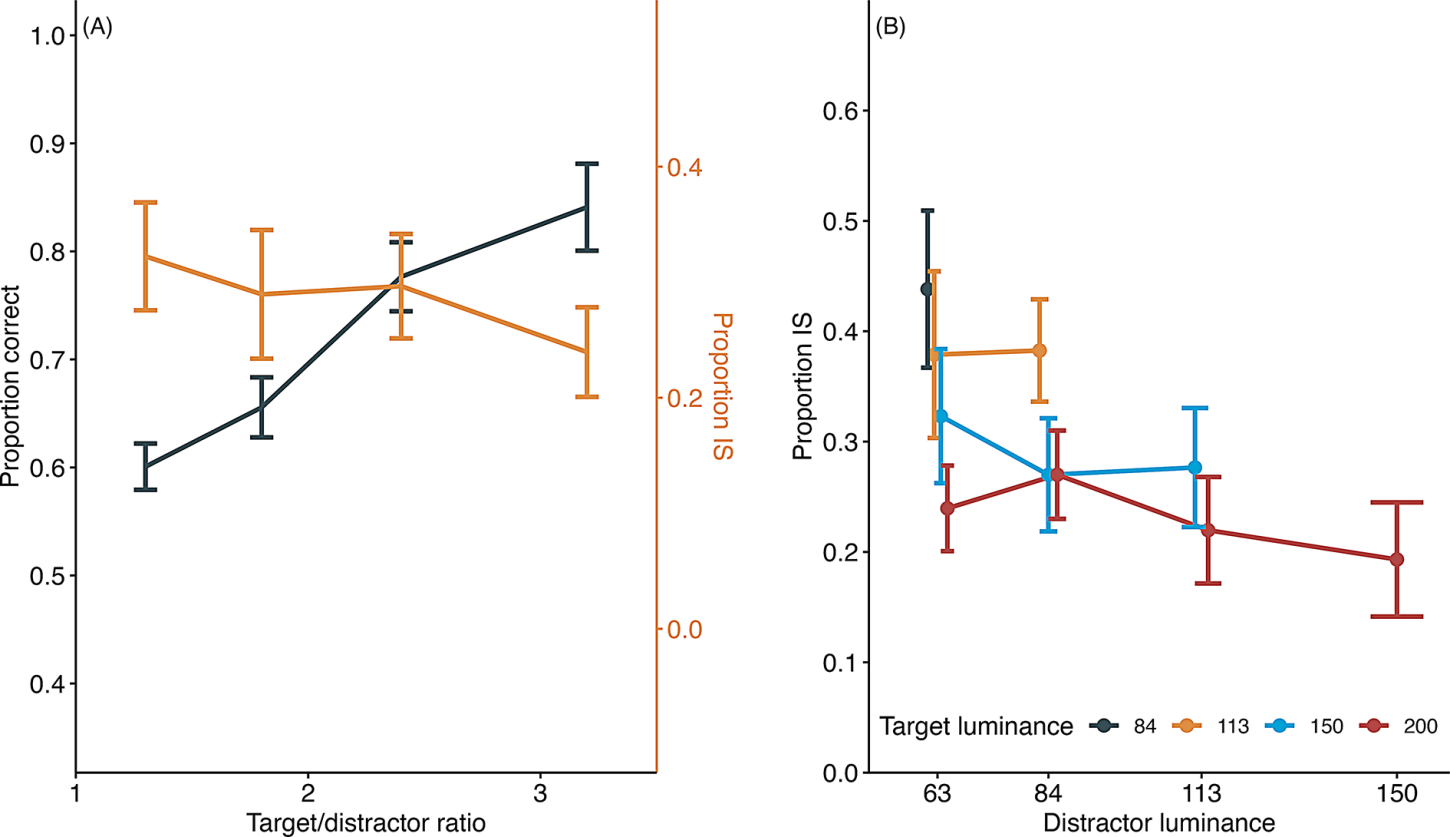
Proportion correct and proportion of information-seeking (IS) in experiment 2. (**A**) The black line represents the proportion correct in baseline trials, and the orange line represents the proportion of IS behavior during information-seeking trials. (**B**) The proportion of IS across ten target-distractor stimulus combinations during information-seeking training in Experiment 2. Error bars indicate the standard error of the mean. Individual data are shown in supplementary file Fig. 2S

Figure 6 illustrates the proportion of correct responses and the rate of information-seeking behavior in the information-seeking trials for each stimulus pair. The proportion of correct responses was higher for stimulus pairs with higher luminance differences, while the rate of information-seeking behavior was lower for these pairs. A GLMM was fitted to the proportion of information-seeking behavior, with luminance ratio as a fixed factor and subjects as a random factor, revealing a significant main effect of luminance ratio (χ^2^(1) = 6.02, *p* =.014).

At first glance, these results support the interpretation that the mice sought information when discriminating between stimulus pairs that were more difficult to discriminate. However, further examination of the data revealed an alternative explanation. As shown in Fig. 6b, the information-seeking rate for the same distractor stimulus was lower when the target stimulus was brighter and had a higher luminance ratio. Conversely, the information-seeking rate decreased for the same target stimulus when the distractor stimulus was brighter, and the luminance difference was slight.

Interestingly, when the luminance of the target stimulus remained constant, there was little change in the rate of information-seeking, even when the luminance of the distractor stimulus increased, and the luminance ratio between the target and distractor stimuli decreased. These results suggest that the mice did not use the luminance difference between stimulus pairs as a cue to search for information, which is inconsistent with the interpretation that metacognitive process drove their information-seeking behavior.

### Test 1

**Fig. 7:**
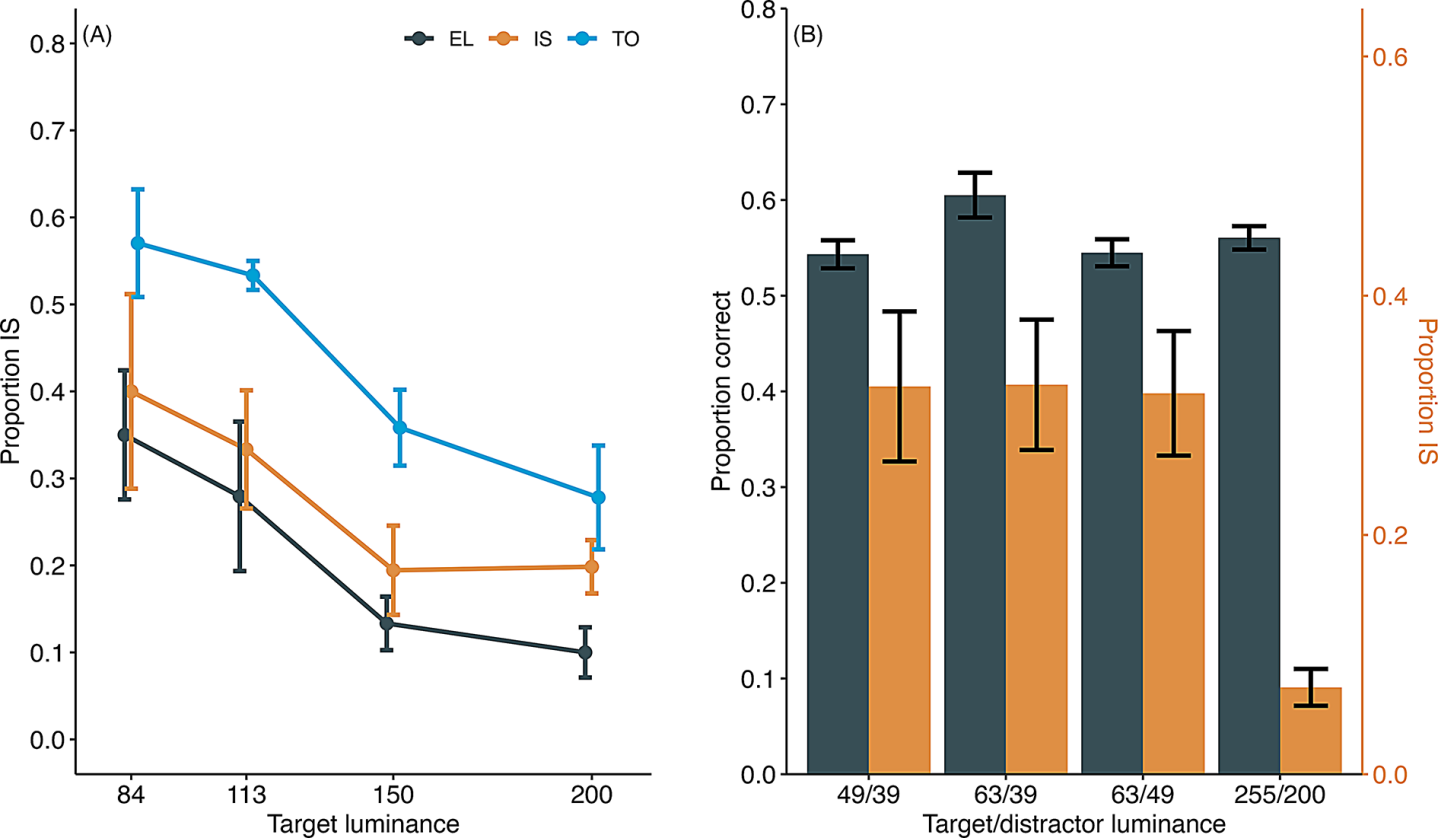
Proportion of information-seeking (IS) behavior in tests 1 and 2 of experiment 2. (**A**) Test 1: EL stands for equiluminant trials, IS for information-seeking trials, and TO for target-only trials. (**B**) Test 2: Proportion correct and proportion of IS for each stimulus pair in test 2. Error bars indicate the standard error of the mean. Individual data are shown in supplementary file Fig. 3S

Figure 7a shows the rate of information-seeking as a function of target luminance. Overall, the rate of information-seeking decreased as the luminance of the target stimulus increased across all three trial types. Notably, regardless of luminance, information-seeking behavior was more frequent in TO trials, in which it was not required, and least frequent in EL trials, in which it was necessary to locate the target stimulus. A GLMM fitted to the proportion of information-seeking responses, with target luminance and trial type as fixed factors and subjects as a random factor, revealed that both main effects were significant (target luminance: χ^2^(1) = 76.21, *p* <.001; trial type: χ^2^(2) = 60.85, *p* <.001).

The mean proportions of correct responses were 0.67, 0.52, and 1.00 in information-seeking, EL, and TO trials, respectively, when the mice did not choose the information-seeking option. If the metacognitive processes drove the information-seeking response, the mice would be expected to choose the information-seeking option more frequently in EL trials and less in TO trials. However, contrary to our predictions, information-seeking rates were highest in TO trials and lowest in EL trials. Additionally, information-seeking rates decreased as the luminance of the target stimulus increased across all trial types. As the luminance of the entire task screen was highest in EL trials and lowest in TO trials, it is likely that the overall screen luminance influenced information-seeking: the darker the screen, the more likely the mice were to seek information, and the brighter the screen, the less likely.

### Test 2

Figure 7b shows the rate of information-seeking for each stimulus pair. The luminance differences between the target and distractor stimuli were nearly identical across all pairs, making the difficulty level consistent for all trials. However, the frequency of information-seeking behavior varied significantly depending on the luminance of the target stimulus. Specifically, information-seeking behavior was observed more frequently in trials with darker target stimuli (63/49, 63/39, 49/39), while it was almost absent in trials with brighter target stimuli (255/200). A GLMM fitted to the proportion of information-seeking responses revealed a significant main effect of the stimulus pair (target and distractor) (χ^2^(3) = 94.71, *p* <.001), and Tukey’s multiple comparisons indicated a significantly lower information-seeking rate with the brightest stimulus pair (255/200) compared to the other three conditions (Table 2). A GLMM fitted to the proportion of correct responses in the baseline condition did not find a significant main effect of the stimulus pair (χ^2^(3) = 4.75, *p* =.19). These results further support the interpretation that luminance served as a cue for information-seeking behavior.

**Table 2:** Results of multiple comparisons of proportion IS between luminance pair conditions

Comparison pair	z value	adjusted *p*-value
49/39–255/200	9.14	< 0.001
63/39–255/200	8.94	< 0.001
63/49–255/200	8.70	< 0.001
63/39–49/39	-0.26	0.99
63/49–49/39	-0.60	0.93
63/49–63/39	-0.34	0.99

In this experiment, we successfully manipulated task difficulty based on the luminance differences between stimulus pairs and effectively shaped the information-seeking behavior of the mice during the discrimination task. However, we found that the information-seeking behavior was not driven by internal cue such as confidence but by environmental cues like luminance. Luminance is a highly salient cue for mice, and they likely used the luminance cue from the entire screen, perhaps because they had been trained to discriminate luminance in the primary task. Therefore, in experiment 3, we investigated whether mice would seek information adaptively when the primary task did not rely on luminance as a discrimination cue.

## Experiment 3: information-seeking behavior in orientation discrimination

In experiment 3, we investigated the information-seeking behavior of mice during an orientation discrimination task. Initially, we trained the mice to discriminate between two lines based on which one was closer to vertical or horizontal. The difference in orientation between the two lines systematically influenced the mice’s discrimination performance. After establishing this baseline, we introduced an information-seeking option during the discrimination task. Finally, we examined the mice’s information-seeking behavior in conditions similar to those in experiment 2.

## Method

### Stimuli

During the initial training phase, the mice were trained to discriminate between horizontal lines (0° orientation) and vertical lines (90° orientation), both measuring 80 pixels (2 cm) in length. However, due to difficulty learning the discrimination, the line length was increased to 120 pixels (3 cm) starting in session 23. Once the mice successfully learned this discrimination, four additional pairs of stimuli were introduced, consisting of lines oriented at 10° and 80°, 20° and 70°, 30° and 60°, and 40° and 50°.

### Procedure

#### Orientation discrimination

The mice were divided into two groups. Half of the mice (T01, T03, and T05) were trained with more vertical lines as the positive stimulus (S+), while the remaining mice (T02, T04, and T06) were trained with more horizontal lines as the S+. All other procedures mirrored those used in the luminance discrimination task of experiment 2. Each daily session consisted of 60 randomly intermixed trials, excluding correction trials, with trials equally divided among the stimulus pairs. Discrimination between horizontal and vertical lines was trained over 30 sessions, followed by an additional 14 sessions during which the four new stimulus pairs were introduced.

### Information-seeking training

After completing the orientation discrimination phase, an information-seeking option was introduced while the mice performed the discrimination task. Each session included the same three types of trials as in experiment 2. In all trials, responses to the target stimulus were reinforced with a food pellet, while responses to the distractor stimulus resulted in a 5-second time-out during which the house light was turned off. Stimulus pairs and target locations were randomized and counterbalanced on a trial-by-trial basis. Each session comprised 20 information-seeking training trials, 20 information-seeking trials, and 20 baseline trials, totaling 60 trials. This training phase lasted for 22 sessions.

### Test 1

In this phase, the same types of test trials used in test 1 of experiment 2 were introduced in addition to baseline and IS trials. As illustrated in Fig. 4, TO trials presented only the target stimulus on either side of the information-seeking option. In Equiorientation (EO) trials, the two stimuli presented to the left and right of the information-seeking option had the same orientation, with one designated as the target stimulus. Each session consisted of 10 TO trials, 20 information-seeking trials, 10 EO trials, and 20 baseline trials for 60 trials. This phase lasted for 21 sessions.

### Test 2

In test 2, the stimuli used in Test 1 were altered by switching from the training S + stimuli to the training S- stimuli in the EO and TO trials. All other aspects of the experiment remained the same as in test 1. Test 2 also lasted for 21 sessions.

## Results and discussion

### Orientation discrimination

**Fig. 8:**
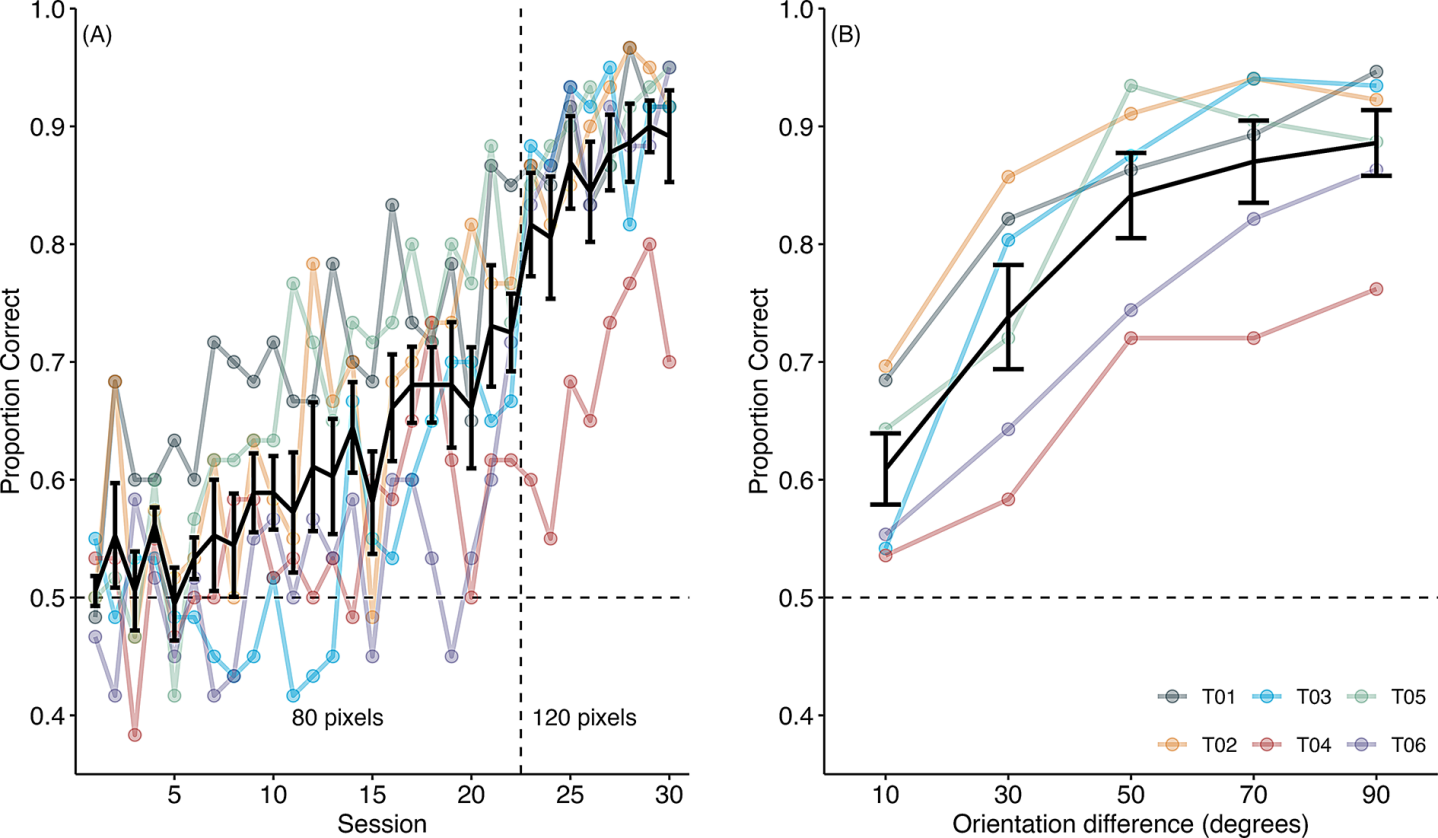
Orientation discrimination in experiment 3. (**A**) Acquisition of horizontal and vertical discrimination. The black line represents the mean percentage correct, while the colored lines show individual performance. Up to session 22, the line segments were 80 pixels in length. From session 23, the line length was increased to 120 pixels to improve accuracy. (**B**) Proportion correct in orientation discrimination. The black line represents the mean percentage correct, whereas the colored lines show individual performance. Error bars indicate the standard error of the mean

Figure 8a shows the proportion of correct responses during the orientation discrimination task over 30 sessions. Initially, all six mice struggled with the task, which led to an increase in line length from 80 pixels to 120 pixels in session 22. After this adjustment, the mice’s discrimination performance improved. Figure 8b presents the proportion of correct responses based on the orientation difference between paired stimuli. A GLMM revealed a significant main effect of orientation difference (Xχ^2^(3) = 274.83, *p* <.001). Both groups of mice—those trained with more vertical lines as the positive stimulus (S+) and those trained with more horizontal lines as S+—demonstrated increased accuracy with higher orientation differences. As a result, data from both groups were combined for further analysis.

### Information-seeking training

**Fig. 9:**
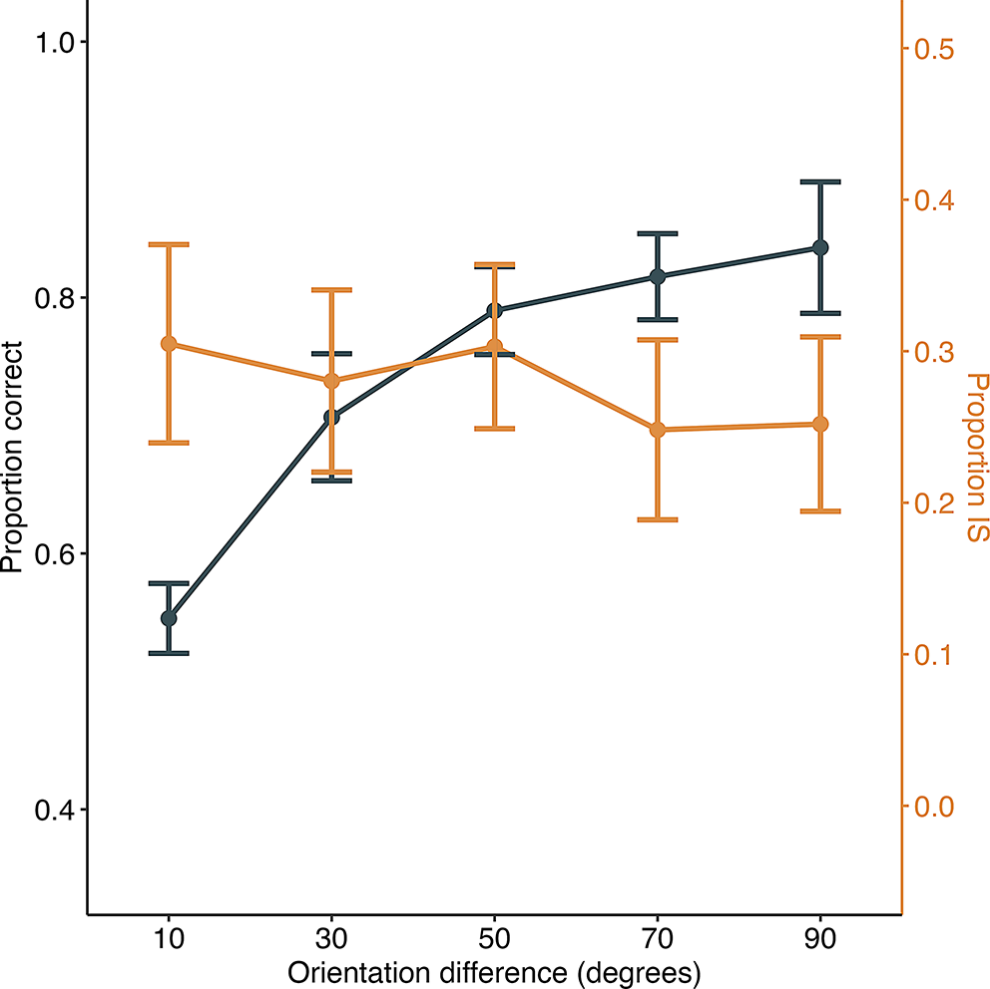
Proportion correct and proportion of information-seeking (IS) in experiment 3. The black line represents the proportion correct in baseline trials, and the orange line represents the proportion of IS behavior during information-seeking trials. Error bars indicate the standard error of the mean. Individual data are shown in supplementary file Fig. 4S

Figure 9 illustrates the proportion of correct responses and the rate of information-seeking during the orientation discrimination task. Trials with higher orientation differences were associated with higher proportions of correct responses, while information-seeking behavior was more frequent during trials with more minor orientation differences. A GLMM showed a significant main effect of orientation difference on the rate of information-seeking (χ^2^(1) = 5.47, *p* =.02), indicating that mice may engage in information-seeking behavior during more challenging trials, similar to the findings in experiment 2.

### Test 1

The mean proportion of correct responses was 1.0 in TO trials and 0.48 in EO trials, with a performance of 0.75 in information-seeking trials. If information-seeking behavior were solely based on task difficulty, we would expect more information-seeking responses in EO trials and fewer in TO trials.

**Fig. 10:**
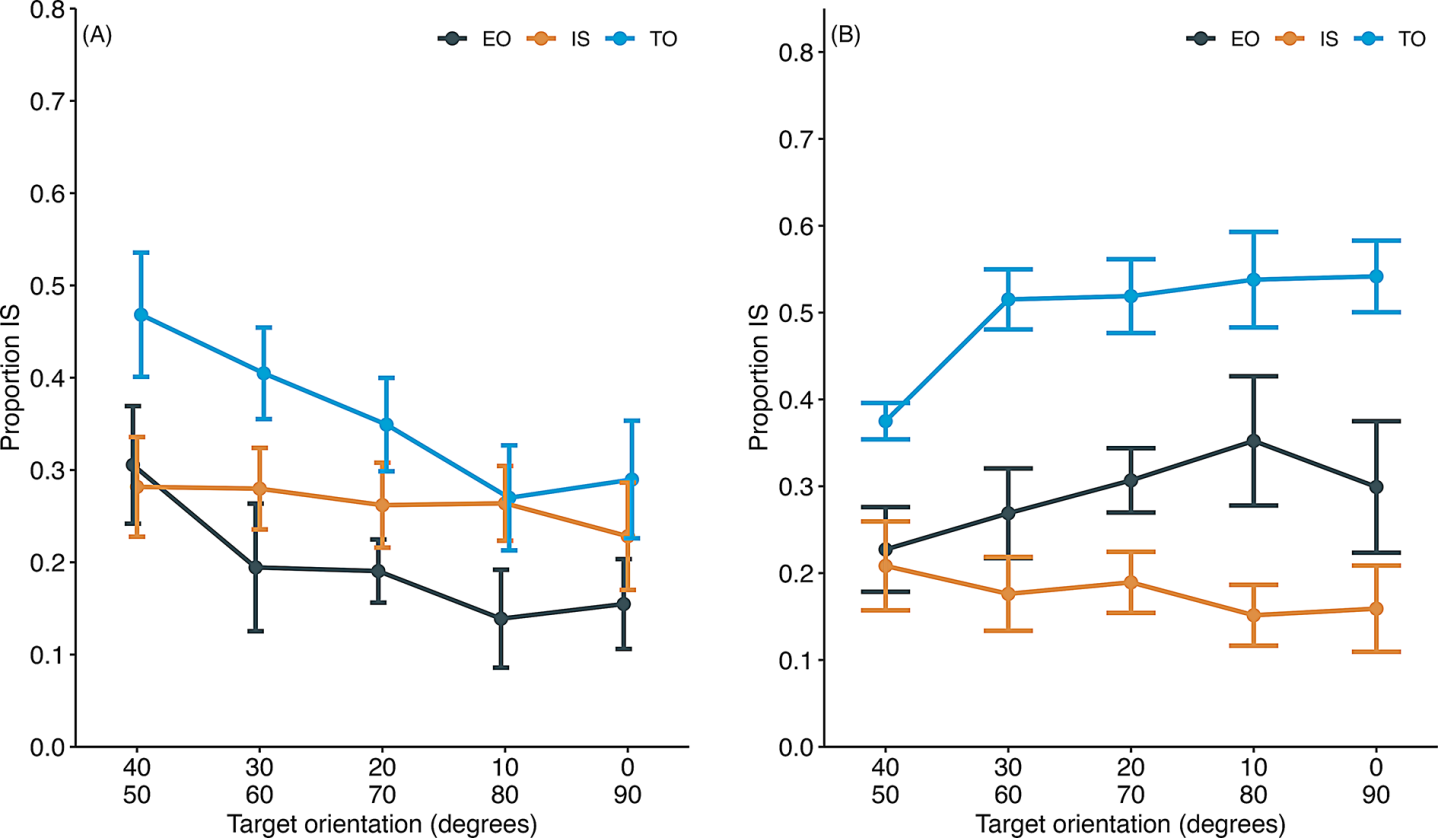
Proportion of information-seeking (IS) behavior in tests 1 and 2 of experiment 3. (**A**) Test 1: EO stands for equiorientation trials, IS for information-seeking trials, and TO for target-only trials. In EO trials, two identical training targets were presented along with the information-seeking option. (**B**) Test 2: EO stands for equiorientation trials, IS for information-seeking trials, and TO for target-only trials. In EO trials, two identical training distractors were presented along with the information-seeking option. Error bars indicate the standard error of the mean. Individual data are shown in supplementary file Fig. 5S

Figure 10a shows that information-seeking rates decreased as orientation differences increased in TO and EO trials. In information-seeking trials, however, this rate was less influenced by orientation differences. A GLMM revealed significant main effects of target orientation (χ^2^(1) = 41.08, *p* <.001) and trial type (χ^2^(2) = 89.10, *p* <.001), as well as a significant interaction between these factors (χ^2^(2) = 12.29, *p* <.01). Interestingly, information-seeking was more prevalent in TO trials than in EO trials, contrary to expectations. This finding suggests that factors other than task difficulty, such as the saliency of S + and the number of choice alternatives, may play a role in influencing information-seeking behavior. When the most salient stimuli (0° or 90°) were presented on both sides in EO trials, they may have attracted the mice to choose either one, reducing the rate of information-seeking. In contrast, with fewer alternatives, TO trials may have increased information-seeking behavior.

### Test 2

Figure 10b illustrates the information-seeking rate across the three test trial types. Consistent with test 1, the highest rate of information-seeking was observed in TO trials, with a higher information-seeking rate in EO trials than information-seeking trials. The influence of target orientation on information-seeking rates was less pronounced compared to test 1. A GLMM revealed significant main effects of target orientation (χ^2^(1) = 4.08, *p* =.04) and trial type (χ^2^(2) = 417.39, *p* <.001), along with a significant interaction between these factors (χ^2^(2) = 22.31, *p* <.001). In EO trials, two negative stimuli (S-) led to a higher proportion of information-seeking responses than information-seeking trials, likely due to the S- avoidance. Additionally, within EO trials, pairs of stimuli with more considerable orientation differences elicited a higher proportion of information-seeking responses, as subjects tended to avoid the S-. Similarly, more considerable orientation differences in stimulus pairs corresponded with a higher proportion of information-seeking responses in TO trials.

## Experiment 4: impact of task height on information-seeking behavior

In order to enable animals to seek information adaptively, it is essential to reconcile the relative costs of task responses and information-seeking behavior. In the tube task, lowering the height of the table on which the tube is placed reduces the occurrence of information-seeking behavior, and adjusting the weight of the tube prevents animals from lifting it excessively (Hampton et al. 2004). Experiment 4 explored how the vertical positioning of stimuli, or task height, influences information-seeking behavior in mice. Increasing task height was hypothesized to raise the response cost, as the mice would need to stand-up higher to respond. We aimed to determine whether this increased effort would result in more frequent information-seeking behavior, similar to previous findings in which response costs influenced decision-making and information-seeking in other tasks.

## Method

### Stimuli

The stimuli in this experiment were the same as those in experiment 3.

### Procedure

This experiment consisted exclusively of IS trials. The task height was manipulated by adjusting the vertical position of the target and distractor stimuli on the screen. Three different heights were tested: the original position (0 pixels), 50 pixels higher, and 100 pixels higher. The information-seeking option remained in its original position at the bottom center of the monitor. All other aspects of the procedure mirrored those used in experiment 3.

Each session included 60 trials, with 20 trials for each stimulus height. The experiment was conducted 5 days a week, lasting for 21 sessions.

## Results and discussion

**Fig. 11:**
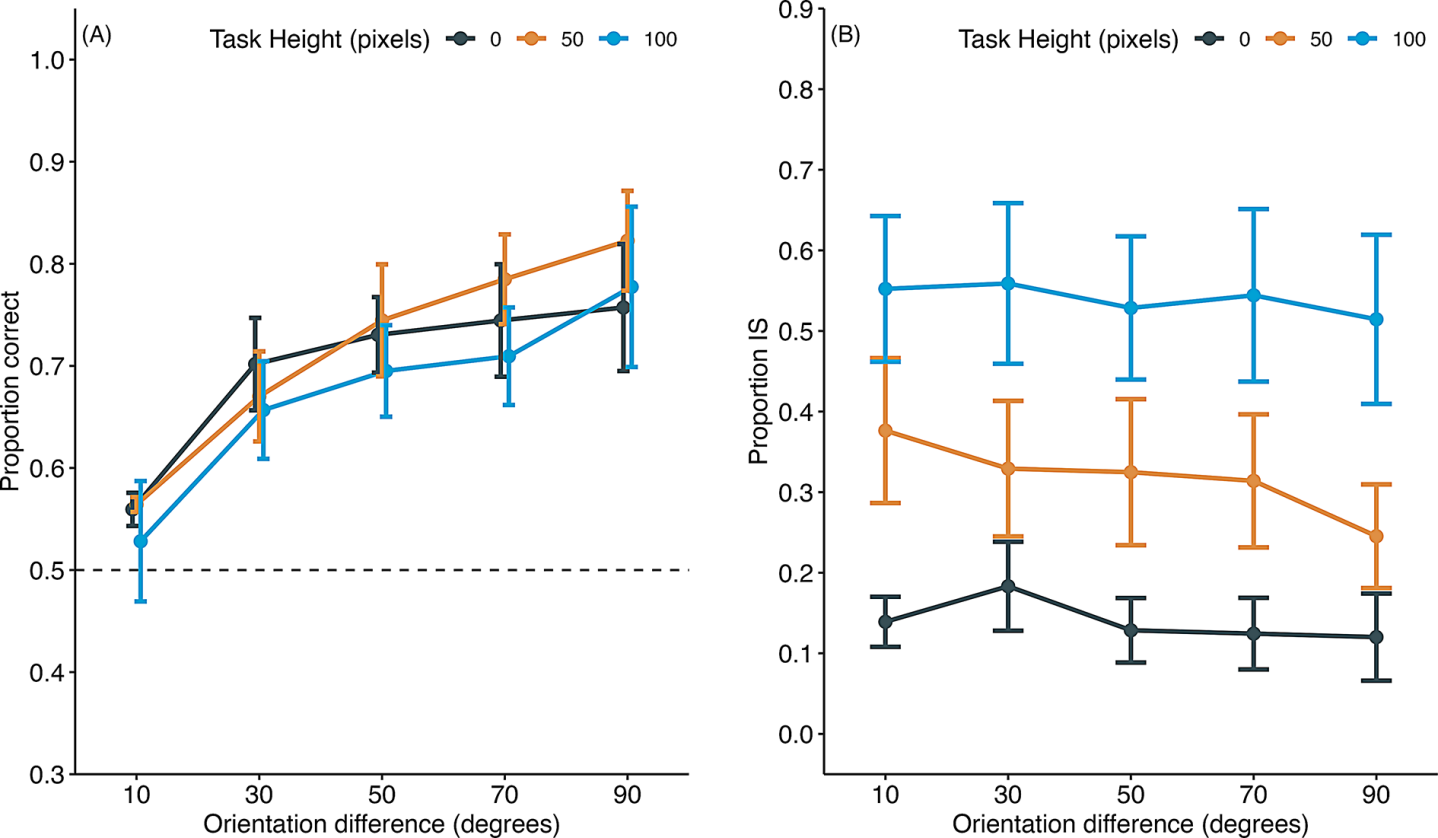
Proportion correct and information-seeking (IS) behavior in information-seeking trials of experiment 4. (**A**) Proportion correct. (**B**) Proportion of IS behavior. Error bars indicate the standard error of the mean. Individual data are shown in supplementary file Fig. 6S

Figure 11a presents the proportion of correct responses on information-seeking trials in which the mouse did not engage in information-seeking behavior. Similar to experiment 3, the proportion of correct responses decreased as the orientation differences between stimulus pairs became small, and this trend was consistent across different target heights. A GLMM was applied to the proportion of correct responses, with orientation difference and target height as fixed factors and subjects as random factors. The analysis revealed a significant main effect of orientation difference (χ^2^(1) = 115.89, *p* <.001). However, the effects of target height (χ^2^(1) = 5.82, *p* =.05) and its interaction with orientation difference (χ^2^(1) = 2.67, *p* =.26) were not significant.

Figure 11b shows the rate of information-seeking as a function of target height. The rate of information-seeking behavior slightly decreased with more considerable orientation differences and increased with higher target positions. A GLMM fitted to the proportion of information-seeking, with orientation difference and target height as fixed factors and subjects as a random factor, revealed significant main effects of both orientation difference (χ^2^(1) = 21.12, *p* <.001) and target height (χ^2^(1) = 859.42, *p* <.001). The interaction between orientation difference and target height was not significant (χ^2^(1) = 5.13, *p* =.08).

These results suggest that mice increased their information-seeking behavior as the response cost of the primary discrimination task increased with target height. However, an alternative interpretation is that the observed increase in information-seeking was due to the relative increase in response cost of the primary task compared to the information-seeking option. These findings demonstrate that the frequency of information-seeking behavior can be experimentally manipulated by adjusting the relative response costs between the primary discrimination task and the information-seeking option.

## Experiment 5: impact of choice alternatives on information-seeking behavior

In order to encourage animals to seek information adaptively, the number of choice alternatives was manipulated in earlier studies. For instance, capuchin monkeys sought information more frequently when the number of choice alternatives was six compared to when it was two (Beran et al. 2016). Rats who did not opt out of the memory test when the number of choice alternatives was two, opted out when the number of choice alternatives was six (Yuki and Okanoya 2017). In experiment 5, we investigated the effect of the number of choice alternatives in the primary task on information-seeking behavior. We hypothesized that information-seeking behavior would more likely occur when there were more choice alternatives.

## Method

### Stimuli

The stimuli used were the same as those in experiments 3 and 4.

Procedure.

The experiment consisted exclusively of information-seeking trials. The number of choice alternatives was two or four, determined on a trial-by-trial basis. The stimuli were arranged horizontally, with 180 pixels of spacing between the centers of the stimuli. The stimuli were presented in two positions near the center of the screen if there were two choice alternatives. The vertical position of both the target and distractor stimuli was set 50 pixels higher than the position of the information-seeking option to facilitate the response to the information-seeking option, as in experiment 4. All other procedural aspects mirrored those used in experiment 3. Each session consisted of 40 trials, with 20 trials per choice alternative condition. The experiment was conducted 5 days a week, continuing until 30 sessions were completed.

## Results and discussion

**Fig. 12:**
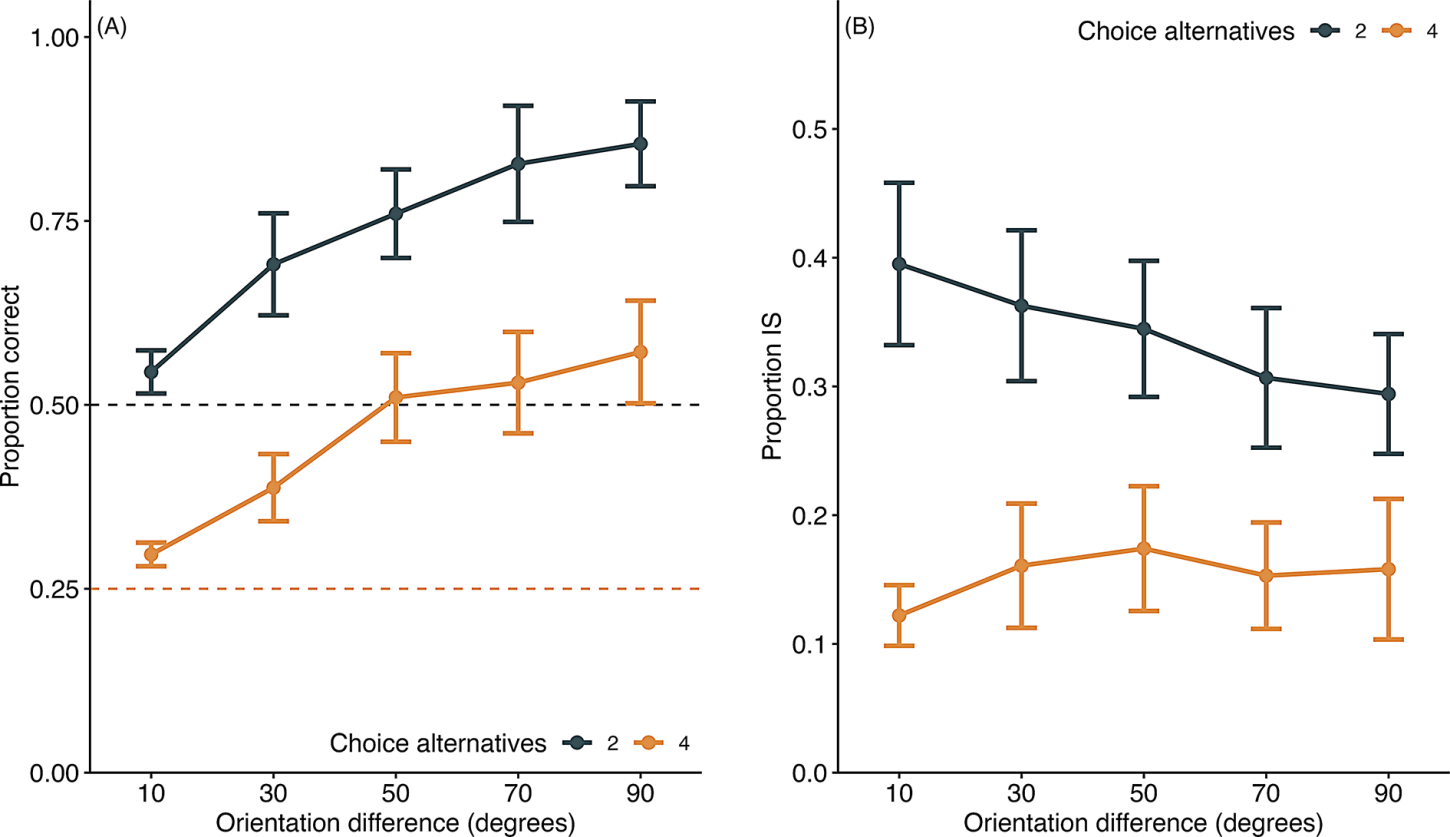
Proportion correct and information-seeking (IS) behavior in information-seeking trials of experiment 5. (**A**) Proportion correct. (**B**) Proportion of IS behavior. Error bars indicate the standard error of the mean. Individual data are shown in supplementary file Fig. 7S

Figure 12a shows the proportion of correct responses as a function of the orientation difference for each number of choice alternatives. The proportion of correct responses was higher with two choice alternatives than four, although it remained well above chance (0.25). Consistent with the findings in experiment 3, the proportion of correct responses decreased as the orientation difference between stimuli became small, a trend observed across both conditions. A GLMM fitted to the proportion of correct responses, with orientation difference and number of choices as fixed factors and subjects as a random factor, revealed significant main effects of orientation difference (χ^2^(1) = 238.65, *p* <.001) and number of choices (χ^2^(1) = 428.04, *p* <.001), as well as a significant interaction between these two factors (χ^2^(1) = 4.28, *p* =.04).

Figure 12b illustrates the rate of information-seeking behavior as a function of the number of choice alternatives. The rate of information-seeking was significantly lower when there were four options compared to two. A GLMM fitted to the rate of information-seeking, with orientation difference and number of choices as fixed factors and subjects as a random factor, showed significant main effects of orientation difference (χ^2^(1) = 8.01, *p* <.01) and number of choices (χ^2^(1) = 337.13, *p* <.001), along with a significant interaction between these factors (χ^2^(1) = 17.53, *p* <.001).

These results suggest mice do not increase information-seeking behavior in response to difficulties in line orientation discrimination. Instead, they are more likely to seek information when there are fewer options, indicating a tendency to engage in information-seeking behavior if the number of alternatives is reduced, thereby simplifying the task environment.

## General discussion

In this series of experiments, we designed two visual discrimination tasks to manipulate task difficulty and investigate information-seeking behavior in mice. One key finding from our experiments is the relationship between task difficulty and information-seeking rate in visual discrimination tasks. Experiment 2 demonstrated that mice sought more information as luminance discrimination became more difficult, as indicated by decreasing luminance differences. Similarly, experiment 3 showed that mice engaged in more information-seeking as the disparity between orientations decreased. This pattern, in which information-seeking increases with task difficulty, aligns with findings from information-seeking experiments in pigeons (Castro and Wasserman 2013). Initially, these findings suggested a potential metacognitive component to information-seeking behavior, implying that mice sought more information when they were less confident about their choices. However, subsequent tests revealed that factors other than internal cues may influence information-seeking behavior (Hampton 2009).

The TO and EL trials in experiment 2 were mainly instrumental in identifying the cues driving information-seeking behavior. Only the target stimulus was presented in TO trials, making the information-seeking response redundant. If internal cues drove information-seeking, it would not occur during TO trials. However, the rate of information-seeking was higher in TO trials, in which the mice could only respond to the target or information-seeking stimulus, compared to information-seeking trials, in which the mice had the option to respond to one of three stimuli. This finding suggests that response competition plays a significant role in information-seeking behavior.

We presented two identical stimuli in EL trials, with one randomly reinforced. We would expect such behavior in EL trials if internal cues were the driving force behind information-seeking. However, the proportion of information-seeking was lower in EL trials than in information-seeking trials, indicating that internal cues are not the primary source of stimulus control for information-seeking behavior. Additionally, target stimuli with a more robust reinforcement history were associated with lower rates of information-seeking in TO and EL trials, further suggesting that reinforcement history influences the rate of information-seeking. While one might be concerned that the mice could have solved this task by simply searching for a stimulus with a particular luminance value, we believe this was not the case. In test 2 of experiment 2, the mice showed similar proportions of correct responses to novel stimulus pairs, suggesting that they compared the two stimuli and selected the brighter one from the pair.

In experiment 3, the TO and EO trials replicated the TO and EL trials findings, reinforcing the interpretation that information-seeking behavior is driven by response competition and reinforcement history rather than internal cues. Additionally, the higher rate of information-seeking observed in EO trials during test 2, in which two identical S- stimuli were presented, further supports the idea that reinforcement history plays a crucial role in shaping information-seeking behavior.

Experiment 4 demonstrated that increasing the response cost by raising the stimulus presentation position led to more frequent information-seeking. This finding is consistent with similar adjustments in the tube task, in which increasing the tube height encourages more information-seeking behavior (Hampton et al. 2004; Marsh and McDonald 2012). Although this experiment did not confirm that internal cues drive information-seeking, it showed that the frequency of information-seeking can be manipulated by altering the relative cost of the discrimination response compared to the information-seeking response.

Experiment 5 found that mice were more likely to seek information when there were fewer alternatives (two) than more alternatives (four). If internal cues were the basis for information-seeking, an increase in alternatives should have led to more information-seeking. However, we observed the opposite: more alternatives led to fewer correct responses and information-seeking rates, further reinforcing that response competition is crucial in driving information-seeking behavior.

In this study, we focused on information-seeking behavior in animals using a computer-based task because the tube task or other variant tasks, another primary method in comparative metacognition research, was not applicable to mice. Both the tube task and computer-based tasks provide valuable insights into animal metacognition, but each has its strengths and limitations (Templer 2022). The tube task is particularly useful for testing novel species, especially when animals can comprehend human actions and their consequences. However, this task is generally limited to species such as primates or canids (Call and Carpenter, 2001; Royka et al. 2020). In contrast, the computer-based task offers the advantage of more controlled experimental conditions, allowing researchers to test multiple hypotheses and carefully analyze the specific cues influencing animal behavior. Through extensive generalization tests, researchers can gain a deeper understanding of metacognitive systems. However, these tasks often require long-term training, which can pose a barrier in certain experimental setups. Rather than choosing between these two approaches, we believe they should be seen as complementary. Researchers can use the tube task or other variant tasks as an initial tool for broad species comparisons or for investigating behavior in less controlled environments, while the computer-based task can be employed for more detailed and systematic exploration of cognitive processes in laboratory settings. By combining both methods, researchers can gain a more comprehensive understanding of animal metacognition.

In summary, the evidence suggests that information-seeking behavior in mice is primarily driven by response competition and reinforcement history rather than by metacognitive control based on internal cues. However, these results do not entirely rule out the possibility that mice may possess metacognitive abilities. While information-seeking behavior has not been conclusively demonstrated in rats (Iwasaki and Taniuchi 2023), evidence suggests that rats can monitor their memory strength (Foote and Crystal 2007; Templer et al. 2017; Yuki and Okanoya 2017). Future research should continue to explore metacognitive monitoring in mice using methodologies similar to those employed with rats.

Despite the challenges, this study successfully established a rodent visual discrimination task that elicited information-seeking behavior. Metacognition has been studied in rhesus monkeys through various manipulated variables in computer-based experiments (Templer 2022), and our study demonstrates that a similar approach is feasible in rodents. In conclusion, it is crucial to consider the development of comparative metacognition. Numerous studies have investigated metacognitive monitoring across species using delayed matching-to-sample tasks to assess decisions to decline difficult tests (Fujita 2009; Goto and Watanabe 2012; Hampton 2001; Inman and Shettleworth 1999; Sutton and Shettleworth 2008). While the tube task remains a standardized tool for information-seeking and species comparison, we hope our methods and findings will help further explore information-seeking behavior in rodents and inspire the development of comparative studies across other species.

## Electronic supplementary material

Below is the link to the electronic supplementary material.

Supplementary Material 1

## Data Availability

All data and scripts for analysis involved in this study have been deposited with OSF. https://osf.io/3nysr/.
