# Correction: Information-seeking in mice (*Mus musculus*) during visual discrimination: study using a distractor elimination paradigm

**DOI:** 10.1007/s10071-025-01990-x

**Published:** 2025-07-26

**Authors:** Yuya Hataji, Kazuhiro Goto

**Affiliations:** 1 Keio University, Tokyo, Japan; 2 Japan Society for the Promotion of Science, Tokyo, Japan; 3 Sagami Women’s University, Kanagawa, Japan

**Correction: Animal Cognition (2024) 27:81**

10.1007/s10071-024-01920-3

In this article the ‘Conflict of Interest’ statement was missing and should have been “Kazuhiro Goto is an editor for the journal and has not taken part in the manuscript’s peer review process.” The original article has been corrected.

