# Supplementary material for: Information-seeking in mice (Mus musculus) during visual discrimination: study using a distractor elimination paradigm
Source: Anim Cogn. 2024 Dec 2;27(1):81. doi: 10.1007/s10071-024-01920-3 (PMC11609126; doi:10.1007/s10071-024-01920-3)

Information-Seeking in Mice (*Mus musculus*) During Visual Discrimination:

Study Using a Distractor Elimination Paradigm

Yuya Hataji & Kazuhiro Goto

Supplementary materials

Fig 1S. Individual data from luminance discrimination in experiment 2. The horizontal axis represents the ratio of target to distractor luminance. Group data are shown in figure 5.

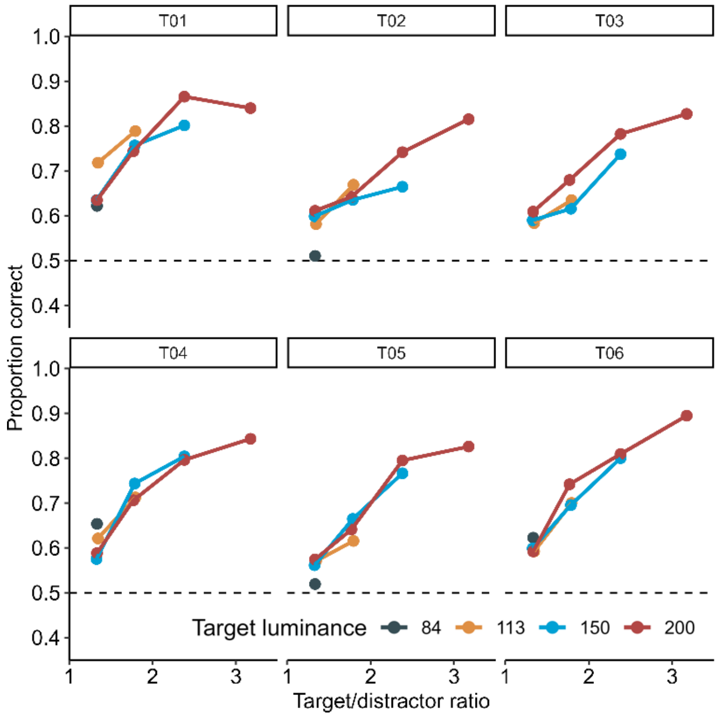

Fig 2S. Individual proportion correct and proportion of information-seeking in experiment 2. (A) The black line represents the proportion correct in baseline trials, and the orange line represents the proportion of information-seeking behavior during IS trials. (B) The proportion of information-seeking across ten target-distractor stimulus combinations during information-seeking training in Experiment 2. Group data are shown in figure 6.

(A) (B)

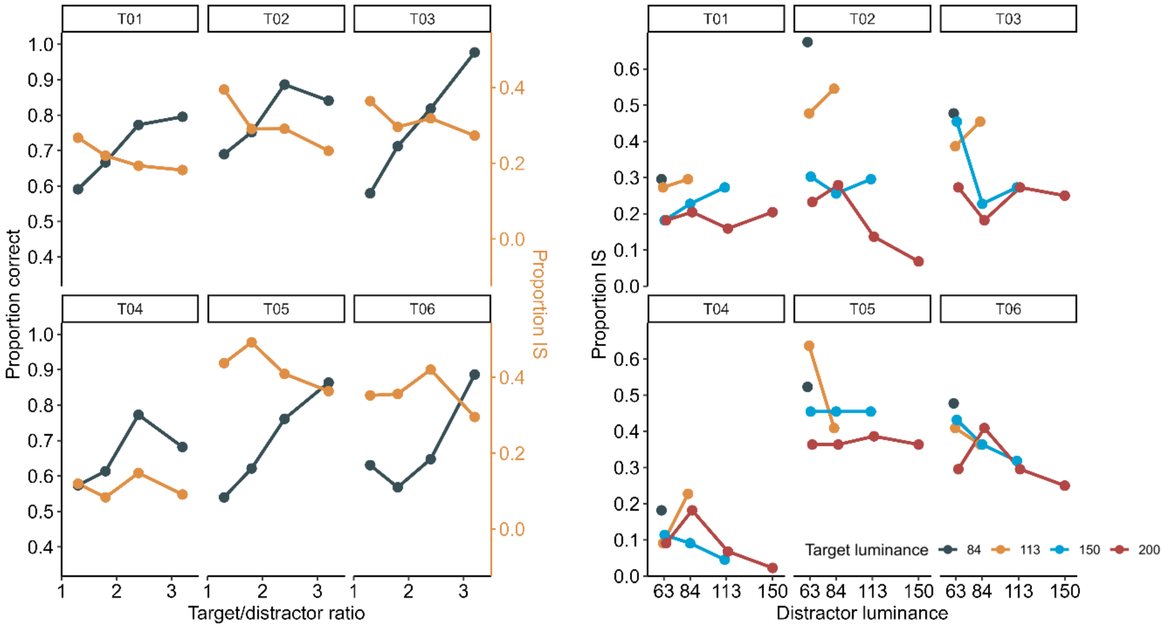

Fig 3S. Individual proportion of information-seeking behavior in tests 1 and 2 of experiment 2. (A) Test 1: EL stands for equiluminant trials, IS for information-seeking trials, and TO for target-only trials. (B) Test 2: Proportion correct and proportion of information-seeking for each stimulus pair in test 2. Group data are shown in figure 7.

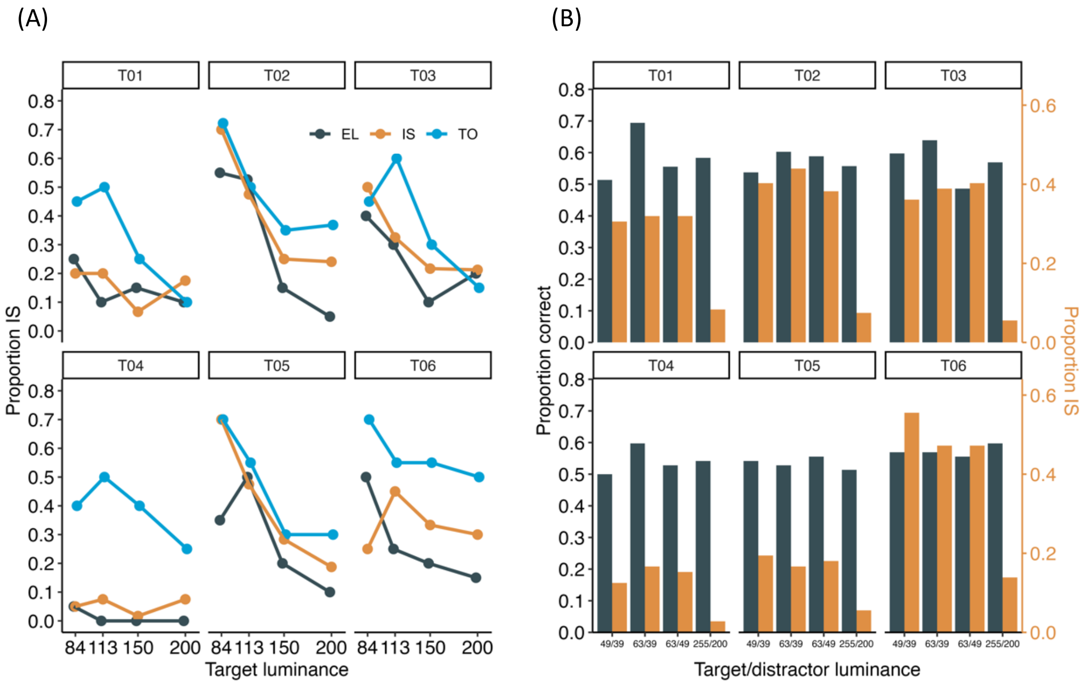

Fig 4S. Individual proportion correct and proportion of information-seeking in experiment 3. The black line represents the proportion correct in baseline trials, and the orange line represents the proportion of information-seeking behavior during IS trials. Group data are shown in figure 9.

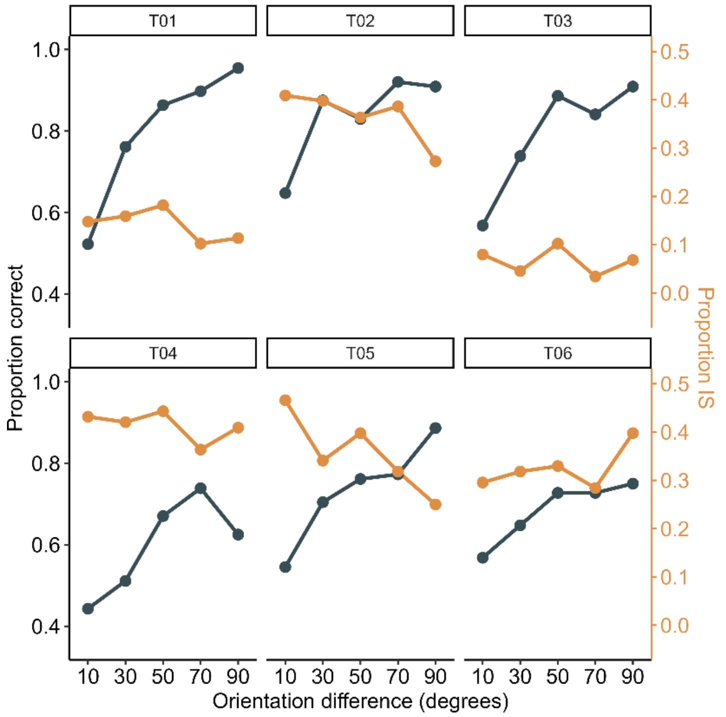

Fig 5S. Individual proportion of information-seeking behavior in tests 1 and 2 of experiment 3. (A) Test 1: EO stands for equiorientation trials, IS for information-seeking trials, and TO for target-only trials. In EO trials, two identical training targets were presented along with the information-seeking option. (B) Test 2: EO stands for equiorientation trials, IS for information-seeking trials, and TO for target-only trials. In EO trials, two identical training distractors were presented along with the information-seeking option. Group data are shown in figure 10.

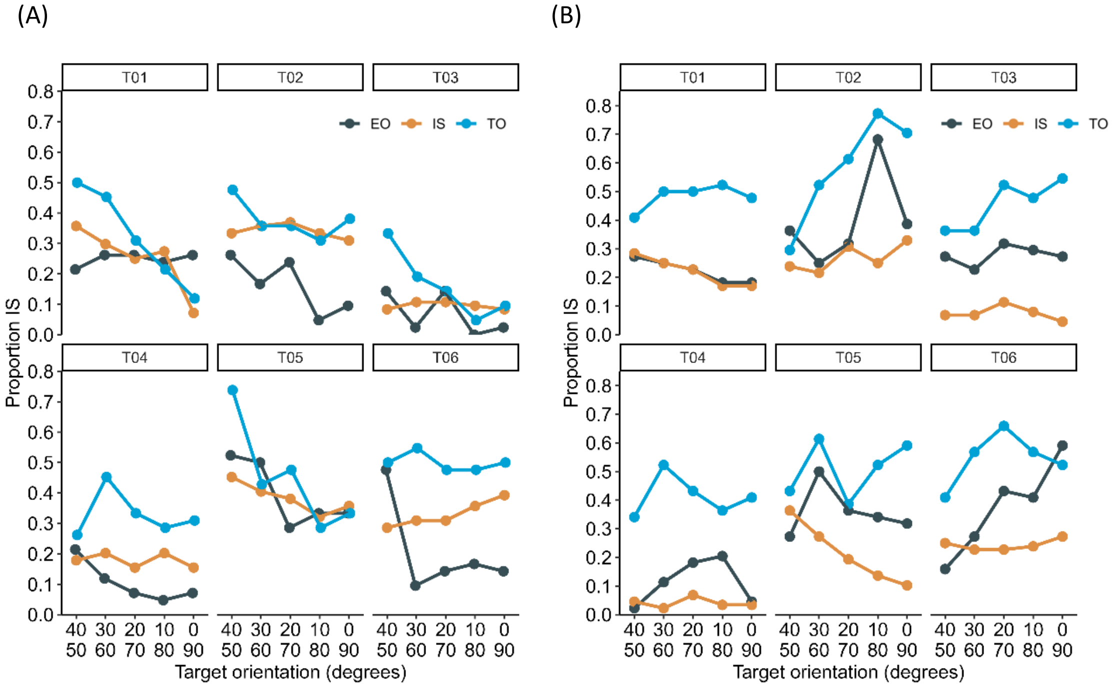

Fig 6S. Individual proportion correct and information-seeking behavior in IS trials of experiment 4. (A) Proportion correct. (B) Proportion of information-seeking behavior. Group data are shown in figure 11.

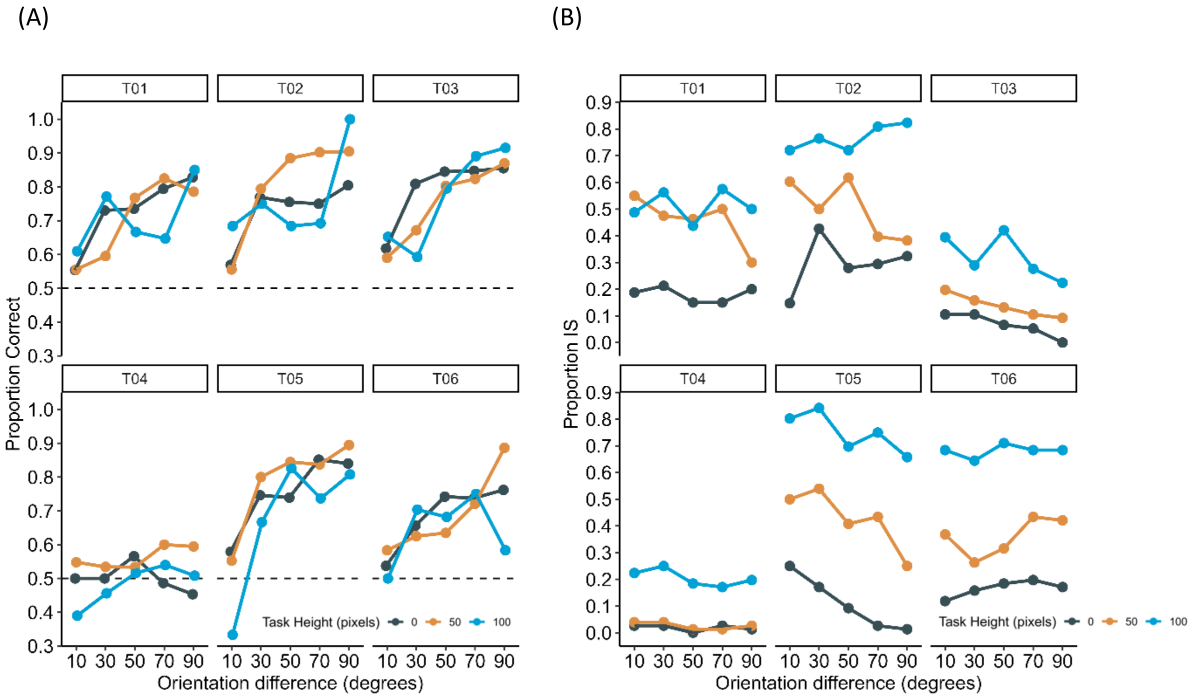

Fig 7S. Individual proportion correct and information-seeking behavior in IS trials of experiment 5. (A) Proportion correct. (B) Proportion of information-seeking behavior. Group data are shown in figure 12.

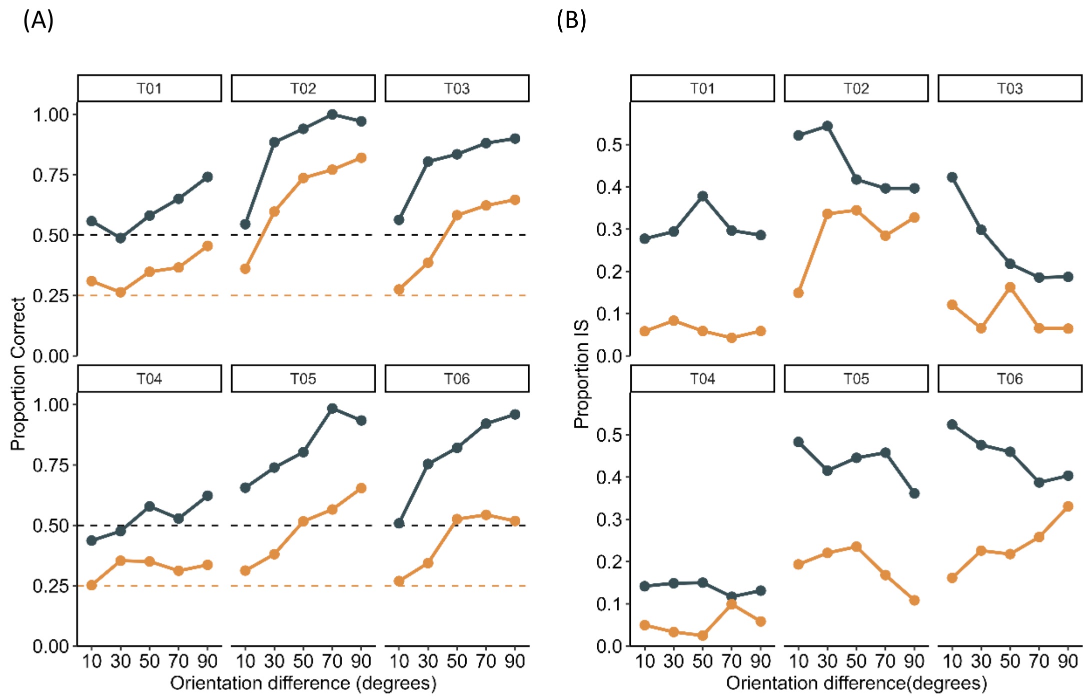

Supplement: Supplementary file 1 — Supplementary Material 1 [file 10071_2024_1920_MOESM1_ESM.pdf]
